# Supplementary material for: Optimizing Axial and Peripheral Substitutions in Si-Centered Naphthalocyanine Dyes for Enhancing Aqueous Solubility and Photoacoustic Signal Intensity
Source: Int J Mol Sci. 2023 Jan 23;24(3):2241. doi: 10.3390/ijms24032241 (PMC9916426; doi:10.3390/ijms24032241)
Supplement: Supplementary file 1 [file ijms-24-02241-s001.zip › ijms-2112268-supplementary.pdf]

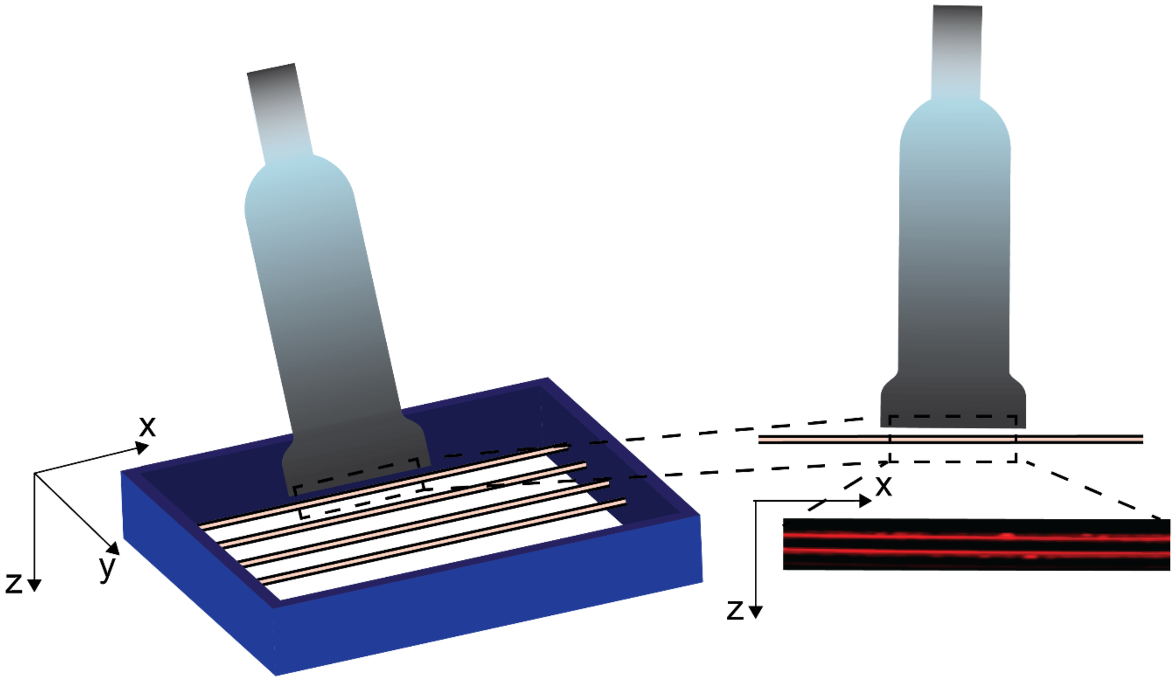

Supplementary Figure S1. Schematic representation of the setup used to record photoacoustic spectra of different dyes in solution. The dyes were resuspended in the desired solvent and injected in polyethylene tubes of 1.4 mm inner diameter and 1.9 mm outer diameter which were subsequently mounted into a custom designed 3D printed box for imaging. The transducer was placed vertically over the tubes for imaging.

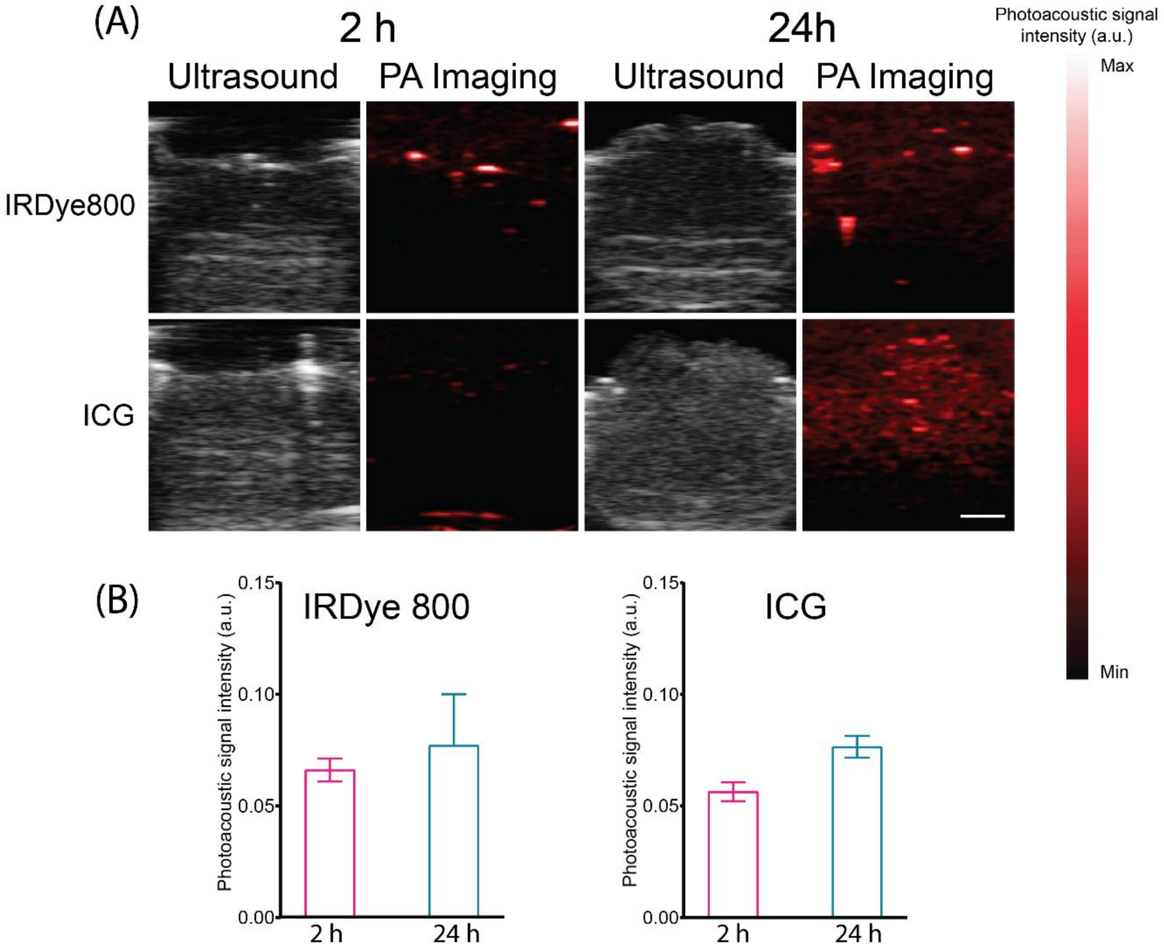

Supplementary Figure S2. Photoacoustic imaging and quantification of photoacoustic signal intensity of IRDye800 and ICG treated Cal 27 cell phantoms. (A) Ultrasound and contrast enhanced photoacoustic images of Cal 27 tumor phantoms created after incubating the cells with IRDye800 (775 nm) and ICG (780 nm) at a concentration of 2.5 μM for either 2 h or 24 h. Phantoms created from gelatin and untreated cells were used as controls. Scale bar represents 1 cm. (B) Quantification of photoacoustic signal intensity from Cal 27 cell phantoms incubated with the IRDye800 and ICG recorded at 775 nm, and 780 nm, respectively. Photoacoustic signal intensity (a.u.) bar is provided in the right.
